# One-Step Synthesis of Ultra-Small RhNPs in the Microreactor System and Their Deposition on ACF for Catalytic Conversion of 4–Nitrophenol to 4–Aminophenol

**DOI:** 10.3390/nano15171375

**Published:** 2025-09-05

**Authors:** Adrianna Pach, Konrad Wojtaszek, Ahmed Ibrahim Elhadad, Tomasz Michałek, Anna Kula, Magdalena Luty-Błocho

**Affiliations:** AGH University of Krakow, Faculty of Non-Ferrous Metals, Al. A, Mickiewicza 30, 30-059 Kraków, Poland; apach@agh.edu.pl (A.P.); kwojtasz@agh.edu.pl (K.W.); elhadad@agh.edu.pl (A.I.E.); tomaszm@agh.edu.pl (T.M.); kula@agh.edu.pl (A.K.)

**Keywords:** one-step synthesis, chemical reduction, ultra-small RhNPs, activated carbon fibers, catalyst, 4-nitrophenol

## Abstract

The rising demand for platinum-group metals, driven by their essential applications in catalysis, energy storage, and chemical conversion, underscores the need to identify new sources for their recovery. Waste solutions originating from industrial processes offer a promising alternative source of noble metals. However, due to their typically low concentrations, effective recovery requires a highly targeted approach. In this study, we present a synthetic waste solution containing trace amount of Rh(III) ions as both a medium for metal ion recovery and a direct precursor for catalyst synthesis. Using a bimodal water–ethanol solvent system, ultra-small rhodium nanoparticles were synthesized and subsequently immobilized onto activated carbon fibers (ACFs) within a microreactor system. The resulting Rh@ACF catalyst demonstrated high efficiency in the reduction of 4-nitrophenol (4-NP) to 4-aminophenol (4-AP), serving as a model catalytic reaction. The Rh@ACF catalyst, containing 4.24 µg Rh per milligram of sample, exhibited notable catalytic activity, achieving 75% conversion of 4-NP to 4-AP within 1 h. Full conversion to 4-AP was also reached within 5 min, but requires extra NaBH_4_ addition to the catalytic mixture.

## 1. Introduction

The search for new sources rich in elements belonging to the platinum group of metals (PGMs) [1] is a crucial topic for two main reasons. The first reason is the increasing demand for these metals due to their extensive use, particularly in catalysis [2], energy storage [3], and conversion [4]. The second reason is the unique properties of PGMs, such as high chemical resistance and catalytic activity, which make them irreplaceable by other compounds. In practice, natural deposits of PGMs are virtually nonexistent. Ores with higher concentrations of these elements are located at significant depths, making their extraction both challenging and costly. As a result, PGMs are primarily sourced from other ores, such as copper and nickel sulfides, where they occur as associated metals [5]. This means that PGMs are present in these ores at very low concentrations and in a highly dispersed form. They are typically accumulated during metallurgical processes—for example, in the copper sulfide processing conducted by KGHM Polish Copper. In KGHM’s technological process, PGMs are obtained in the form of slime after copper electrorefining. Subsequently, metals are separated and recovered step by step in the following order: silver (Ag), gold (Au), and a palladium–platinum mixture (Pd-Pt). Alternatively, secondary sources offer a viable route for PGM recovery [6]. Special attention should be given to industries such as metallurgy, petroleum [2], pharmaceuticals [7], and chemistry, where catalysts are frequently used. These catalysts, whether in metallic form (nano- to microscale) or as various compounds, can accumulate in waste solutions (aqueous, organic, etc.), which could serve as an excellent source for nanometric catalyst production. Nowadays, the recovery of PGMs from industrial processes is realized by, e.g., electrodeposition–redox replacement [8,9], adsorption [10], pyrometallurgy [11], hydrometallurgy, and biometallurgy [12], indicating high process efficiency. However there has been no information given about the properties of the recovered materials nor have there been any studies implying their possible applications.

In our previous research, we demonstrated that it is possible to utilize low concentrations of different metal ions as a source for catalyst production. For this purpose, we employed various catalyst carriers [13,14,15] and used a microreactor [14] system for catalyst optimization. In contrast, this study focuses on the previously unexplored rhodium and the application of a bimodal solvent system (water–ethanol) for the synthesis of ultra-small Rh catalysts. A motivation for this study is also the fact that existing procedures for RhNP synthesis are based on high-temperature treatment, mostly using organic solvents that require special treatment and/or have harmful effects on the environment and living organisms. Although the described methods for producing rhodium nanoparticles make it possible to obtain particles with diverse morphologies and potential catalytic application [16,17], these processes are so complex that their industrial application remains unlikely. Consequently, efforts are directed toward developing synthesis methods that utilize simple, minimally toxic reagents, proceed within relatively short timeframes, and require low energy input. Recently, Li et al. [18] described a procedure for environmentally friendly, room temperature synthesis of Rh nanoparticles that potentially meets the outlined requirements. The use of a mixture of ethanol and an aqueous sodium hydroxide solution as a solvent, combined with the addition of poly (vinyl pyrrolidone) (PVP), facilitates rapid complex formation followed by the reduction of metal ions to rhodium nanoparticles (RhNPs) in a batch reactor. For catalytic testing, the RhNPs synthesis process was modified by replacing the stabilizing agent, i.e., PVP with a catalyst support (Vulcan XC-72 carbon), within the reaction mixture. After synthesis, the RhNPs were either collected by centrifugation or filtered in the case of RhNPs deposited on XC-72 carbon, and subsequently washed to remove residual impurities. The obtained catalyst was successfully evaluated as an electrocatalyst, demonstrating higher activity compared to commercial rhodium catalysts.

In contrast, this study employed a microreactor system for both the synthesis of RhNPs and their deposition onto activated carbon fibers used as the catalyst support. This integrated approach consolidates the process into a single step, enabling the automation of catalyst production and facilitating scalability. Furthermore, conducting the synthesis within a closed system significantly minimizes solvent evaporation, thereby reducing the risk of inhalation exposure and enhancing overall safety for the experimenter. The resulting Rh@ACF catalyst was successfully applied for the catalytic reduction of 4-nitrophenol (4-NP) to 4-aminophenol (4-AP), as a model catalytic reaction [19,20,21,22,23].

## 2. Experimental

### 2.1. Reagents

As a metal precursor, a stock solution containing RhCl_3_·xH_2_0 in 0.1 M HCl, with a concentration of 0.055 M, was used. In the experiments, a volume of 31 µL of stock’s solution was diluted into 10 mL of water (0.17 mM). This solution was always freshly prepared before the experiments. As a complexing solution, the mixture of ethanol (96%, Chempur, Karlsruhe, Germany, p.a.) and an aqueous solution of 0.2 M NaOH (POCH, p.a.) mixed at volumetric ratio 1:1 was used (ET/H_2_O).

As a reducing agent, a basic solution of 0.02 M of sodium borohydride was applied. For this purpose, 0.04 g of powder was dissolved in 0.2 M NaOH, which protected NaBH_4_ from its decomposition in aqua media.

In order to activate the carbon fibers, 88 mL of 95% H_2_SO_4_ (POCH, p.a.) and 58 mL of 99% HNO_3_ (Merck, p.a.) were added to a 250 mL beaker containing the CFs (carbon fibers). Then, KClO_3_ (Roth, Karlsruhe, Germany, 99% p.a.) was added to the mixture in a total amount of 23 g. In the final step, the fibers were washed with 5% HCl (PureLand, Bridgeport, NJ, USA, p.a.). The detailed procedure for the preparation of the activated carbon fibers is described in Section 3.1 (Activation of catalyst carrier).

For the FT-IR analysis, 0.2 g of anhydrous KBr (Merck, Darmstadt, Germany) was used as the matrix material. The appropriate samples, including CF, ACF, and Rh@ACF (each in the amount of 0.001 g), were mixed with KBr and pressed into pellets. A pure KBr pellet was used as a background reference.

For the catalytic test, reagents like sodium borohydride (p.a., POCH, Gliwice, Poland) and 4-nitrophenol (spectrophotometric grade, Sigma Aldrich, Taufkirchen, Germany) were used. These compounds were prepared by dissolving proper amounts of the powders in the volumetric flasks to obtain stock solutions with concentrations of 500 µM 4-NP and 40 mM NaBH_4_. The 4-nitrophenolate solution was prepared in the batch reactor by mixing 0.75 mL of 4-NP stock solution and 0.375 mL of 40 mM NaBH_4_ and with the addition of 1.875 mL of deionized water (for a total volume 3 mL per sample).

### 2.2. Methods

#### 2.2.1. UV-Vis Spectrophotometry

UV-Vis spectrophotometry (Shimadzu, Tokyo, Japan), operating within a wavelength range of 190–900 nm and equipped with both a thermostatic and reference cell, was employed for the analysis of colloidal rhodium as well as for monitoring the conversion of 4-nitrophenol to 4-aminophenol. For this purpose, approximately 3 mL of the solution was transferred into a quartz cuvette (Helmah, path length: 1 cm) and placed in the spectrophotometer holder. Water was used as the reference solution.

#### 2.2.2. MP-AES Analysis

The concentration of the obtained rhodium chloride solution was determined using MP-AES (microwave plasma–atomic emission spectroscopy, MP-AES 4200, Agilent, Santa Clara, CA 95051, USA). This method involves spectrometric measurement with excitation in a microwave nitrogen plasma. The instrument operates with nitrogen (from a generator) as the carrier gas. The determination process requires device calibration. To achieve this, a set of calibration solutions was prepared with rhodium concentrations of 0 ppm, 0.5 ppm, 1 ppm, 5 ppm, 10 ppm, 20 ppm, and 40 ppm. The standards were obtained by diluting a stock solution (1000 ppm in 10% HCl, PlasmaCAL, Villebon-sur-Yvette, France) with 1 M nitric acid (POCH, p.a., Gliwice, Poland). The calibration procedure involved measuring the spectrum of these prepared standards. The device’s software generated a calibration curve based on the measured values, with a linear regression fit of R^2^ = 0.99995. It is essential to ensure that the sample’s concentration falls within the range of the calibration standards. If the measured concentration exceeds this range, the solution must be diluted accordingly. In this case, the tested rhodium solution was diluted 100-fold, and this dilution was accounted for by the device software during the measurement. The result was obtained in ppm (mg/L) and then converted to molar concentration, yielding 0.055 M RhCl_3_.

#### 2.2.3. High-Resolution TEM and STEM Observations

The morphology and size of the as-deposited products were characterized using a JEOL JEM-ARM200F NEOARMex high-resolution transmission electron microscope (JEOL Ltd., Tokyo, Japan) (HRTEM) operated at 200 kV under both TEM and STEM modes. Imaging was conducted under bright-field multibeam conditions, as well as using a Segmented Annular All-Field (SAAF) detector. For sample preparation, 100 µL of the RhNP-containing solution was drop-cast onto a copper grid coated with a 30 nm carbon film. The grid was then placed on filter paper to remove excess solvent and left to dry at room temperature (20 °C).

#### 2.2.4. SEM Analysis (RhNPs@ACF)

The distribution of RhNPs on the carbon substrate was examined using a Hitachi SU-70 Schottky (Hitachi High-Tech Corporation, Tokyo, Japan) field-emission scanning electron microscope (SEM). Imaging was carried out using both secondary electron (SE) and backscattered electron (BSE) modes. The chemical composition of the deposited products was confirmed by energy-dispersive X-ray spectroscopy (EDS). For sample preparation, a small amount of the dried catalyst was affixed to the SEM holder using carbon tape.

#### 2.2.5. XPS Analysis

The XPS analyses were carried out in a PHI VersaProbeII Scanning XPS system (Physical Electronics (PHI), Chanhassen, MN, USA) using monochromatic Al Kα (1486.6 eV) X-rays focused to a 100 µm spot and scanned over the area of 400 µm × 400 µm. The photoelectron take-off angle was 45° and the pass energy in the analyzer was set to 117.50 eV (0.5 eV step) for survey scans and 46.95 eV (0.1 eV step) to obtain the high-resolution spectra of Rh 3d, Si 2p, C 1s, O 1s, Na 1s, and Cl 2p regions. A dual-beam charge compensation with 7 eV Ar+ ions and 1 eV electrons was used to maintain a constant sample surface potential regardless of the sample conductivity. All XPS spectra were charge-referenced to the unfunctionalized, saturated carbon (C-C) C1s peak at 285.0 eV. The operating pressure in the analytical chamber was less than 4 × 10^−9^ mbar. Deconvolution of the spectra was carried out using PHI MultiPak software (v.9.9.3). The spectrum background was subtracted using the Shirley method. The information depth of the XPS analysis, within the geometry of spectrometer, can be estimated at about 5 nm.

#### 2.2.6. Fourier-Transform Infrared (FT-IR) Analysis

Fourier-Transform Infrared (FT-IR) analysis was conducted using a Nicolet 380 spectrometer (Thermo Fisher Scientific, Waltham, MA, USA) in transmission mode. Samples were prepared by homogenously grinding 0.001 g of carbon fibers (before activation, after activation, and after Rh adsorption) with 0.2 g of anhydrous potassium bromide (KBr, Merck, Darmstadt, Germany) in an agate mortar. The mixture was compressed into 10 mm diameter pellets under a pressure of 10 MPa using a hydraulic press. Prior to sample measurement, a background scan was performed using a pure KBr pellet to account for atmospheric interference. Spectra were acquired over the wavenumber range of 4000–500 cm^−1^.

#### 2.2.7. Catalytic Tests

The ready-to-use Rh@ACF catalyst, synthesized in the microreactor system and dried in an oven at 60 °C for 1 h, was introduced into the 4-nitrophenolate (4-NPe) solution. Each tested sample contained 0.001 g of Rh@ACF and 3 mL of the 4-NPe solution.

## 3. Results and Discussion

### 3.1. Activation of Catalyst Carrier

Carbon fibers were activated according to the method presented by Staudenmaier [24] and Muszyński [25], whereby the ratio of the acid mixture to the solid phase was increased twice, due to the large volume of dispersed fibers. Commercially available carbon fibers (TohoTenax, Tokio, Japan) were cut into lengths of less than 5 mm. Then 2.5 g of cut fibers together with a Teflon magnetic dipole were placed in a 250 mL beaker placed in a water bath placed on a magnetic stirrer (IKA Werke, Breisgau, Germany). Next, 88 mL of concentrated sulfuric acid (POCH, p.a.) and 58 mL of concentrated, fuming nitric acid (Merck, p.a.) were added to the fibers. Stirring was turned on at 200 RPM. After 5 min of stirring, potassium chlorate (Roth, 99% p.a.) was added in portions of 3–4 g. Each portion was added with a minimum interval of 10 min to avoid overheating the solution, which could negatively affect the activation process and lead to a violent or explosive reaction. Due to the high viscosity of the mixture, it was additionally stirred using a glass rod. After adding a total of 23 g of potassium chlorate, the mixture was left for 6 days with stirring. After this time, the color of the solution changed from yellow to green. The mixture was carefully poured into 2 L of distilled water and then filtered on a paper filter. The fibers on the filter were washed with 250 mL of 5% HCl (PureLand, p.a.) and then with distilled water until the pH of the rinsing was neutral. The filter with carbon fibers was dried in a laboratory dryer (Dry-Line, VWR, Radnor, PA, USA) for 24 h at 70 °C. The carbon fibers obtained in this way were transferred to an amber glass container and used for further experiments. Interestingly, some of the obtained fibers were compacted during activation, obtaining the form of pellets in the shape of a wheat grain and with a relatively high density. The compaction probably occurred as a result of wrapping by a rotating magnetic dipole. Only non-compacted fibers were used for the experiments in this work.

### 3.2. Setup Used for Rhnps Synthesis and Catalyst Deposition on ACF

**Figure 1:**
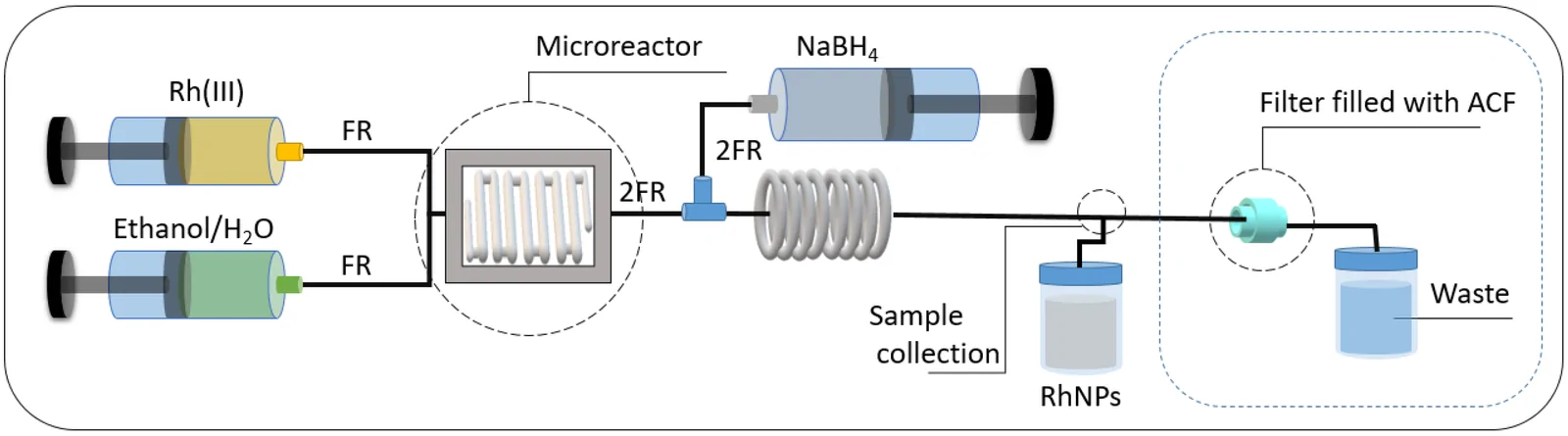
Setup used for RhNP synthesis and their further deposition on activated carbon fibers (ACFs). Main microreactor system components: pumps, glass microchip (V = 250 µL), PTFE microcapillary, filter filled with catalyst as metal trap. Notation: FR—flow rate of solution stream containing Rh (III) ions and ET/H_2_O; 2FR—flow rate of the solution stream containing NaBH_4_ and reagents at the microreactor outlet. Conditions: the temperature of the microchip 30 °C, FR = 0.125 mL/min, 2FR = 0.25 mL/min. Solutions used in experiments: aqueous solution containing Rh (III) ions; mixture of the ethanol 96% and H_2_O (containing 0.2 M NaOH) in volumetric ratio 1:1; solution containing 0.02 M NaBH_4_ in 0.2 M NaOH.

The setup for RhNPs synthesis and catalyst deposition on carrier (ACF) trapped in the filter is shown in Figure 1.

The streams of Rh (III) and ET/H_2_O were pumped via a medical pump (Hawkmed HK-400, Shenzhen Hawk Medical Instrument Co., Ltd., Shenzhen, China) to the glass microreactor chip (V = 250 μL, Dolomite, St Neots, UK) heated up to T = 30 °C. In the microreactor chip, reagents were mixed, allowing the formation of the complex to take place. Based on previous studies (a sample of the kinetic curve is shown in the SM, Figure S1), the process of complex formation requires 1 min at 30 °C. Accordingly, the flow rate of the streams containing the metal precursor and complexing agents (ET/H_2_O) was set to FR = 0.125 mL/min. Next, the formed complex (Rh (III) and ET/H_2_O) was combined with a stream containing NaBH_4_ at T = 20 °C, with a doubled flow rate of 2FR = 0.25 mL/min, in the mixer. The reacting mixture was then directed into a microcapillary loop with a length of 82 cm and an inner diameter of 1.24 mm to ensure the complete reduction of the metal precursor before reaching the catalyst carrier trap. The obtained RhNPs were analyzed spectrophotometrically (Section 3.3) both before and after passing through the filter, and additional characterization was performed using microscopy, XPS, and IR spectroscopy (Section 3.4, Section 3.5, Section 3.6 and Section 3.7).

### 3.3. UV-Vis Analysis

**Figure 2:**
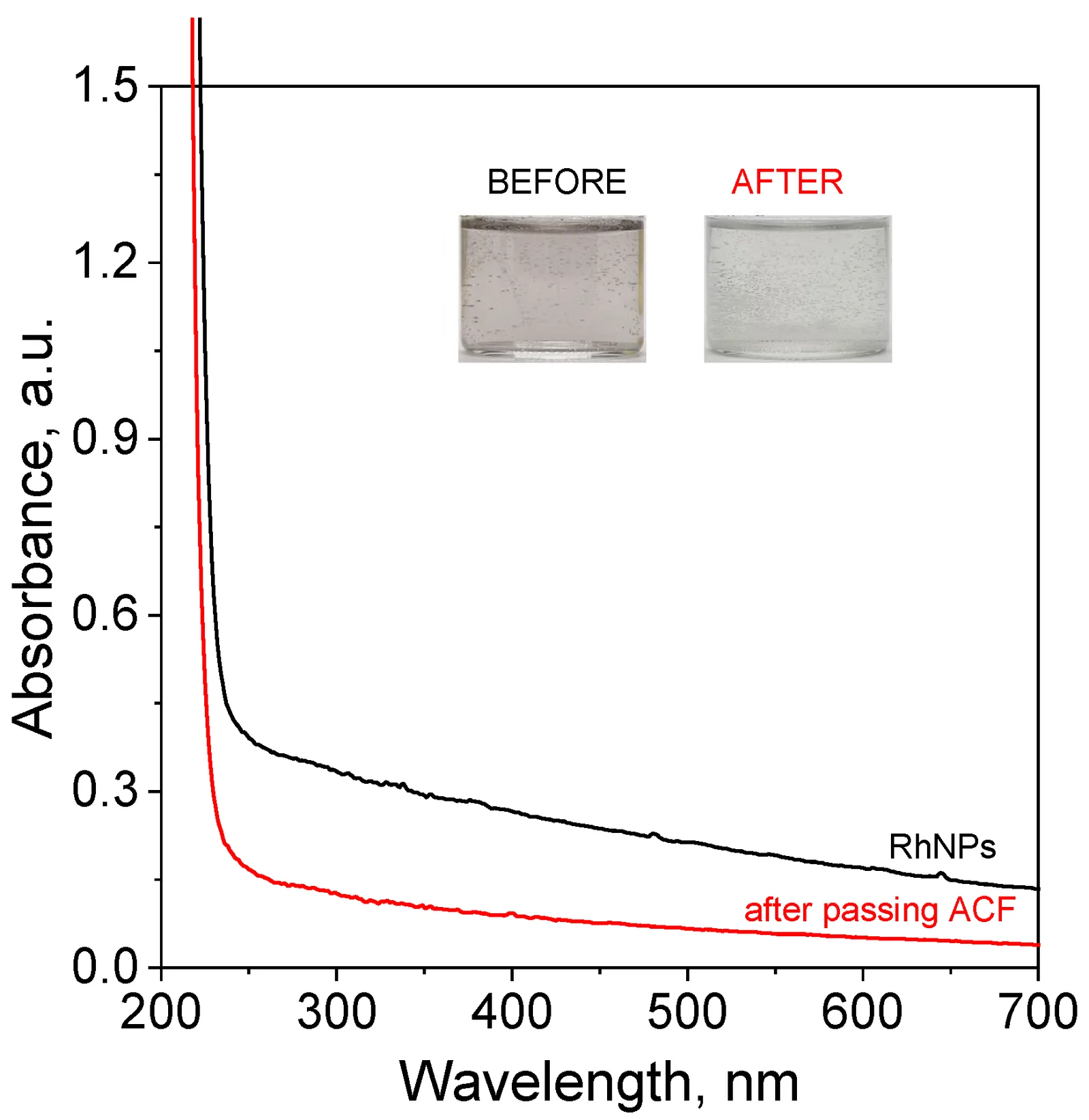
The UV-Vis spectra of solutions containing RhNPs synthesized in the microreactor system before and after passing through the catalyst trap (ACFs). Conditions: C_0, Rh(III)_ = 0.17 mM, mixture of ET/H_2_O (0.82 M for ethanol and 0.1 M for NaOH) (volumetric ratio of ethanol to H_2_O equals 1:1), C_0, NaBH4_ = 0.02 M (in 0.2 M of NaOH), m_ACF_ = 0.0103 g, V_RhNPS_ = 10 mL, T = 30 °C (in the microreactor); synthesis and deposition of RhNPs on ACFs were carried out at 20 °C.

The obtained catalyst, both before and after passing through the filter, as schematically illustrated in Figure 1, was analyzed using spectrophotometry. The observed color differences, along with the recorded spectra, confirmed a reduction in the amount of the metallic phase after the sample passed through the filter containing ACFs (Figure 2).

It is also interesting that the produced RhNPs in the solution enhanced the decomposition of NaBH_4_, leading to a strong hydrogen evolution (visible bubbling in the vials containing the reacting mixture reflects the release of gaseous products, Figure 2). It is important to highlight that highly basic conditions were applied specifically to prevent premature decomposition of sodium borohydride. The observed hydrogen generation under these conditions confirms the strong catalytic activity of the produced RhNPs. This phenomenon is likely related to the extremely small size of the synthesized catalyst. To confirm the morphology of RhNPs, microscopy analysis was conducted using HRTEM (Section 3.4) and SEM (Section 3.5).

### 3.4. TEM and STEM Analysis of RhNPs Synthesized in the Microreactor System (Before Passing Through the Catalyst Trap Containing ACFs)

**Figure 3:**
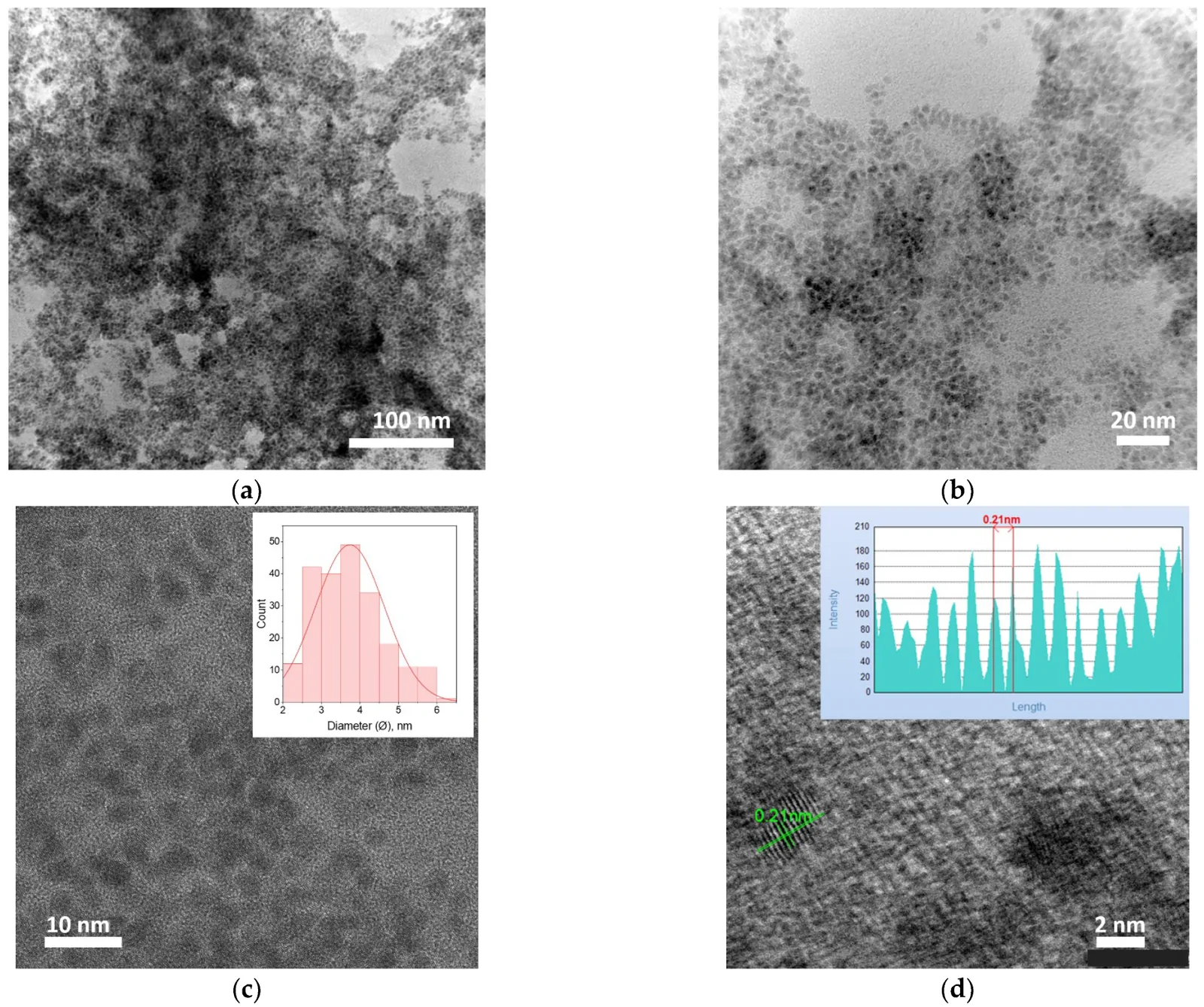
(**a**–**c**) TEM and (**d**) STEM-SAAF images of RhNPs synthesized in the microreactor system. The insets in Figure 3c,d show the mean diameter of the particles and the lattice fringe profile and the measured interplanar spacing of the Rh (111) planes, as determined using JEOL SightX software, respectively. Conditions: C_0, Rh(III)_ = 0.17 mM, mixture of ET/H2O (0.82 M for ethanol and 0.1 M for NaOH) (volumetric ratio of ethanol to H_2_O equals 1:1), C_0, NaBH4_ = 0.02 M (in 0.2 M of NaOH), T = 30 °C (process of complex formation was carried out in the microreactor chip); synthesis of RhNPs was carried out at 20 °C in the microcapillary.

The morphology of the Rh-based catalyst was characterized using both TEM and STEM, as shown in Figure 3. These imaging techniques provided detailed insights into the structural and morphological features of the synthesized Rh nanoparticles (RhNPs).

The TEM and STEM analyses reveal that the RhNPs exhibit a relatively narrow size distribution, with an average particle diameter of approximately 3.0 ± 0.5 nm (Figure 3c,d), indicating a high degree of control over nanoparticle synthesis in the microreactor system. The nanoparticles are predominantly spherical, with well-defined boundaries and minimal size variation across the observed sample area. At lower magnification (Figure 3a), some degree of agglomeration is evident; however, higher magnification images (Figure 3b–d) demonstrate that the majority of RhNPs remain well-dispersed, suggesting a favorable colloidal stability under the synthesis conditions employed. The segmented annular all-field SAAF-STEM images further confirm the homogeneity of the nanoparticle distribution and provide atomic-level contrast that supports the presence of crystalline domains. Lattice fringe analysis, particularly in the inset of Figure 3d, reveals interplanar spacings consistent with the (111) planes of face-centered cubic (fcc) Rh, highlighting the crystalline nature of the synthesized nanoparticles. This combination of uniform morphology, small particle size, and high dispersion is particularly advantageous for catalytic applications, as it maximizes surface-to-volume ratio and ensures a large number of accessible active sites. These morphological characteristics are therefore expected to significantly enhance the catalytic efficiency and stability of the RhNPs under reaction conditions.

### 3.5. SEM Analysis of the RhNPs Synthesized and Deposited on Activated Carbon Fibers

**Figure 4:**
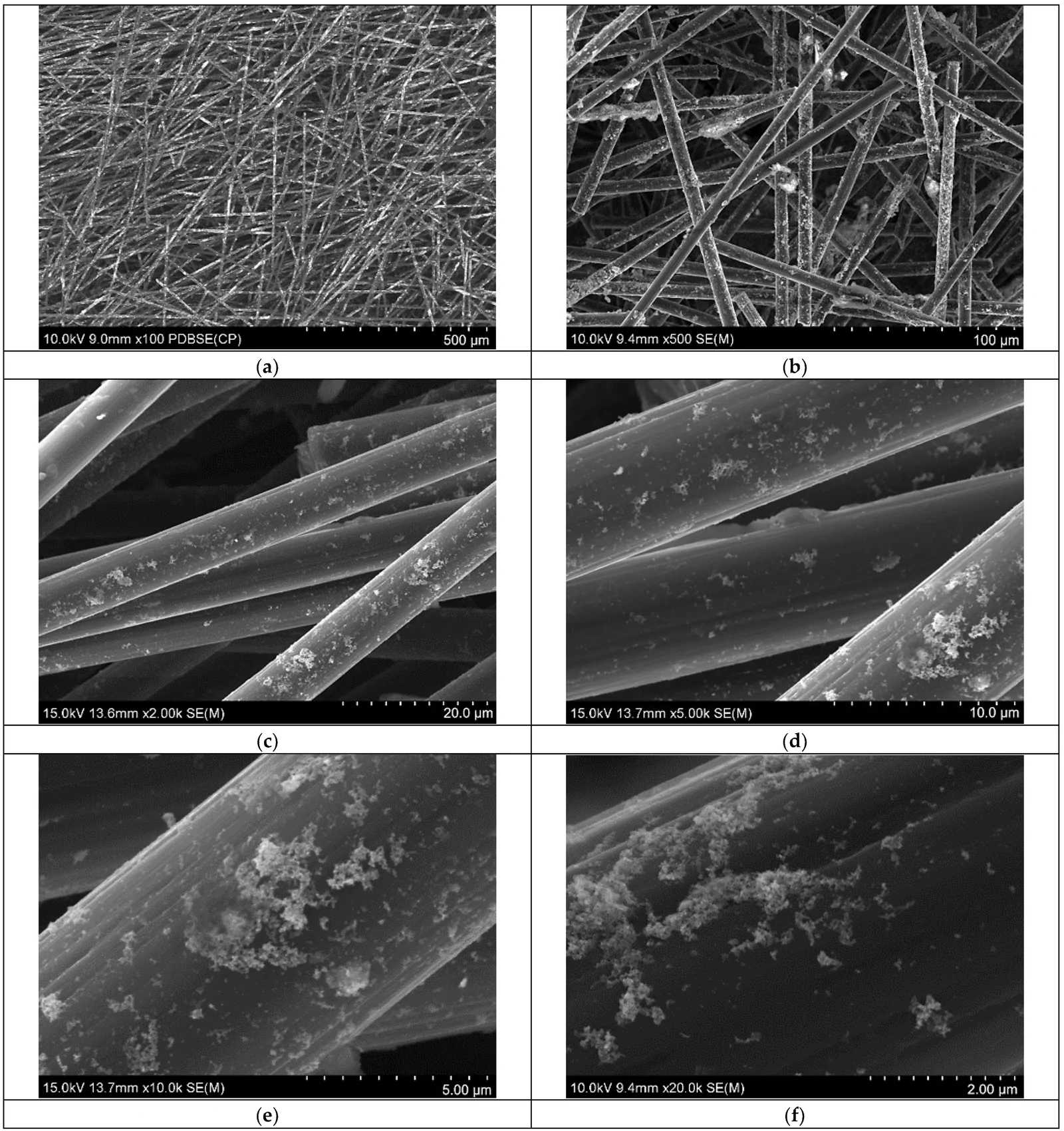
SEM analysis of activated carbon containing RhNPs as a result of synthesis and metal deposition on catalyst carrier carried out in the microreactor system. Sample displayed at various magnifications (**a**–**f**). Conditions: C_0, Rh(III)_ = 0.17 mM, mixture of ET/H_2_O (0.82 M for ethanol and 0.1 M for NaOH) (volumetric ratio of ethanol to H_2_O equals 1:1), C_0, NaBH4_ = 0.02 M (in 0.2 M of NaOH), m_ACF_ = 0.0103 g, V _RhNPS_ = 10 mL, T = 30 °C (process of complex formation was carried out in the microreactor chip); synthesis and deposition of RhNPs on ACFs were carried out at 20 °C in the microcapillary.

Following synthesis, the Rh-based catalyst was deposited onto activated carbon fibers (ACFs) embedded within the filter trap section of the microreactor system (Figure 1). This integration allows for efficient immobilization of the catalyst onto a surface support, facilitating downstream catalytic applications. To confirm the successful deposition of the metallic Rh phase onto the ACF surface, SEM analysis was performed. The resulting micrographs, presented in Figure 4, provide morphological evidence of RhNP presence and their distribution across the fibrous carbon support.

The SEM results confirmed that the activated carbon fibers were uniformly coated with the Rh-based catalyst. Low- to medium-magnification images (Figure 4a–c) show a continuous and homogeneous coverage of the fibrous surface, indicating effective deposition of the catalyst onto the ACF matrix. At higher magnification (Figure 4d–f), the presence of both nanoparticle aggregates and smaller Rh clusters becomes evident. These observations suggest partial aggregation of RhNPs during the immobilization process, while also indicating that a significant fraction of the nanoparticles retained their nanoscale dimensions. However, due to the resolution limits of the SEM instrument, individual ultra-small Rh nanoparticles could not be clearly resolved. The EDS analysis confirmed the presence of Rh on the activated carbon surface (see SM, Section S2). Moreover, the sample contains various elements, including oxygen, sodium, silica, and sulfur (see SM, Table S1), which may be associated with surface groups formed during carbon fiber activation, as well as with adsorbed unreacted ions.

### 3.6. FT-IR Analysis of CF, ACF and Rh@ACF

**Figure 5:**
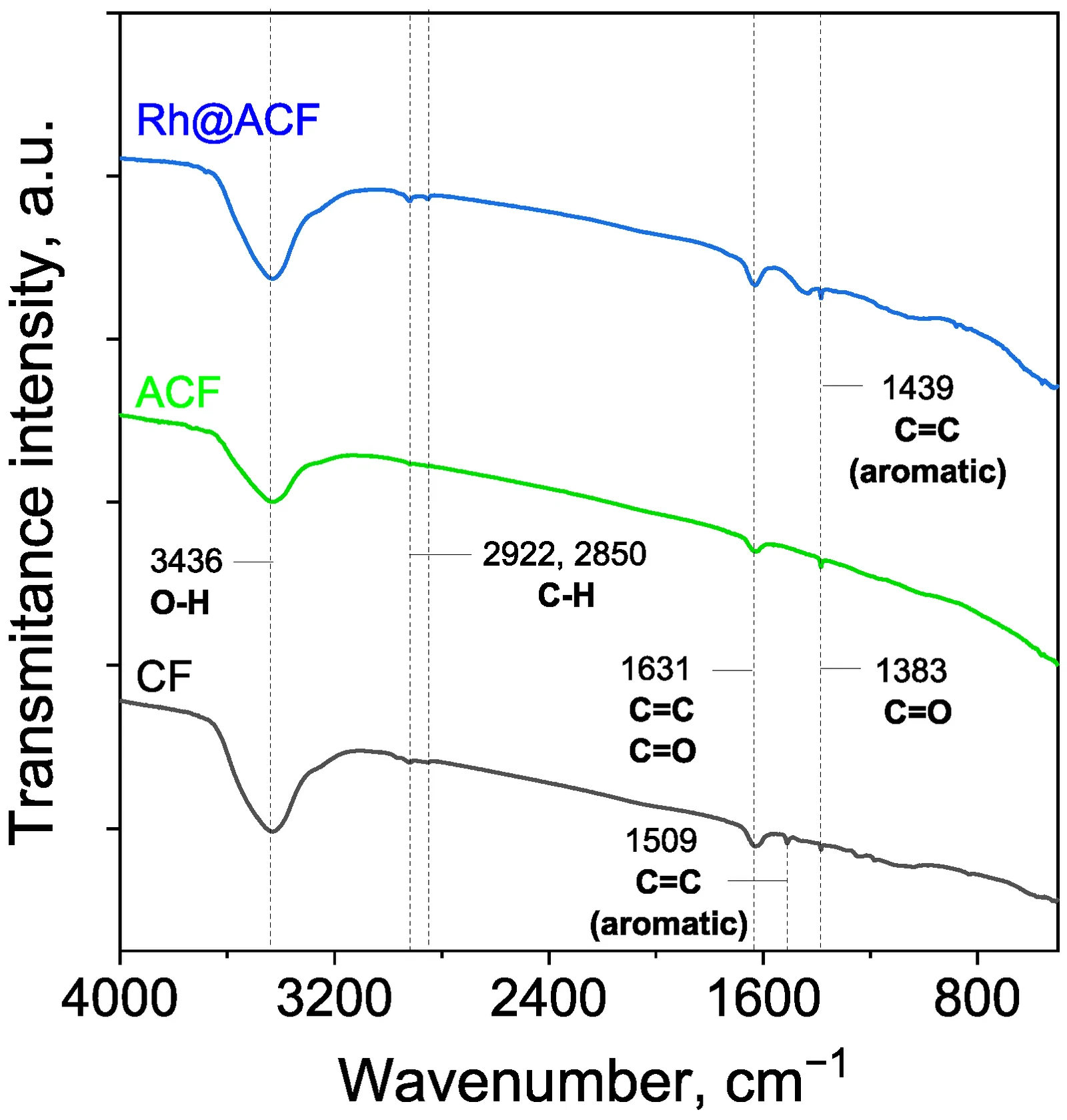
FT-IR spectra of the carbon fiber samples: CF—carbon fibers; ACF—activated carbon fibers; Rh@ACF—catalyst.

FT-IR spectroscopy is a widely applied technique for identifying functional groups and analyzing surface chemistry in carbon-based materials [26]. By measuring the vibrational modes of molecular bonds, FT-IR provides valuable information about the chemical composition and potential modifications arising from treatments such as activation or metal loading. In the context of carbon fiber analysis, it serves as an effective tool to characterize changes in surface functionalities that may influence material performance [27]. The FT-IR spectra obtained for the analyzed samples are presented in Figure 5.

The FT-IR spectra of the carbonaceous materials revealed several characteristic peaks, indicating the presence of various functional groups commonly associated with carbon-based structures. Peaks consistently observed across all samples include those at 3436, 2922, 2850, 1631, and 1383 cm^−1^, while notable differences appeared at 1509 and 1439 cm^−1^, which were exclusive to specific samples.

The broad absorption band centered at 3436 cm^−1^ is attributed to O–H stretching vibrations, typically arising from hydroxyl groups or adsorbed water on the carbon surface [28]. This band was present in all samples but showed a noticeably lower intensity in ACF compared to the other materials, suggesting a reduced surface concentration of hydroxyl groups, potentially due to surface modification. The peaks at 2922 cm^−1^ and 2850 cm^−1^ are attributed to C–H stretching vibrations [29], and are generally linked to the presence of residual organic matter or aliphatic groups. A prominent band at 1631 cm^−1^ is observed in all samples and is associated with either C=C stretching vibrations or C=O stretching in carboxyl groups [30]. The 1509 cm^−1^ peak appeared only in the CF sample, and is attributed to aromatic C=C stretching [28]. Meanwhile, the 1439 cm^−1^ band was observed only in the Rh@ACF sample, and has also been associated with aromatic C=C stretching [31]. Finally, the 1383 cm^−1^ peak, found in all samples, is attributed to the presence of carboxylic C=O groups [32].

Interestingly, despite the presence of rhodium in the Rh@ACF sample, no distinct peaks corresponding to Rh–O vibrations were observed in the FT-IR spectra. Reports in the literature suggest that rhodium oxides typically exhibit a characteristic absorption band at around 550 cm^−1^ [33,34], which is absent in this case. This absence may be attributed to several factors. Firstly, the amount of Rh loading could be too low for its oxide signals to be detected by FT-IR, which has limited sensitivity to low concentrations of inorganic species. Secondly, the rhodium may exist predominantly in its reduced metallic state (Rh^0^), which is IR-inactive due to the lack of dipole moment changes during vibrational modes.

### 3.7. XPS Analysis of Rh@ACF

**Figure 6:**
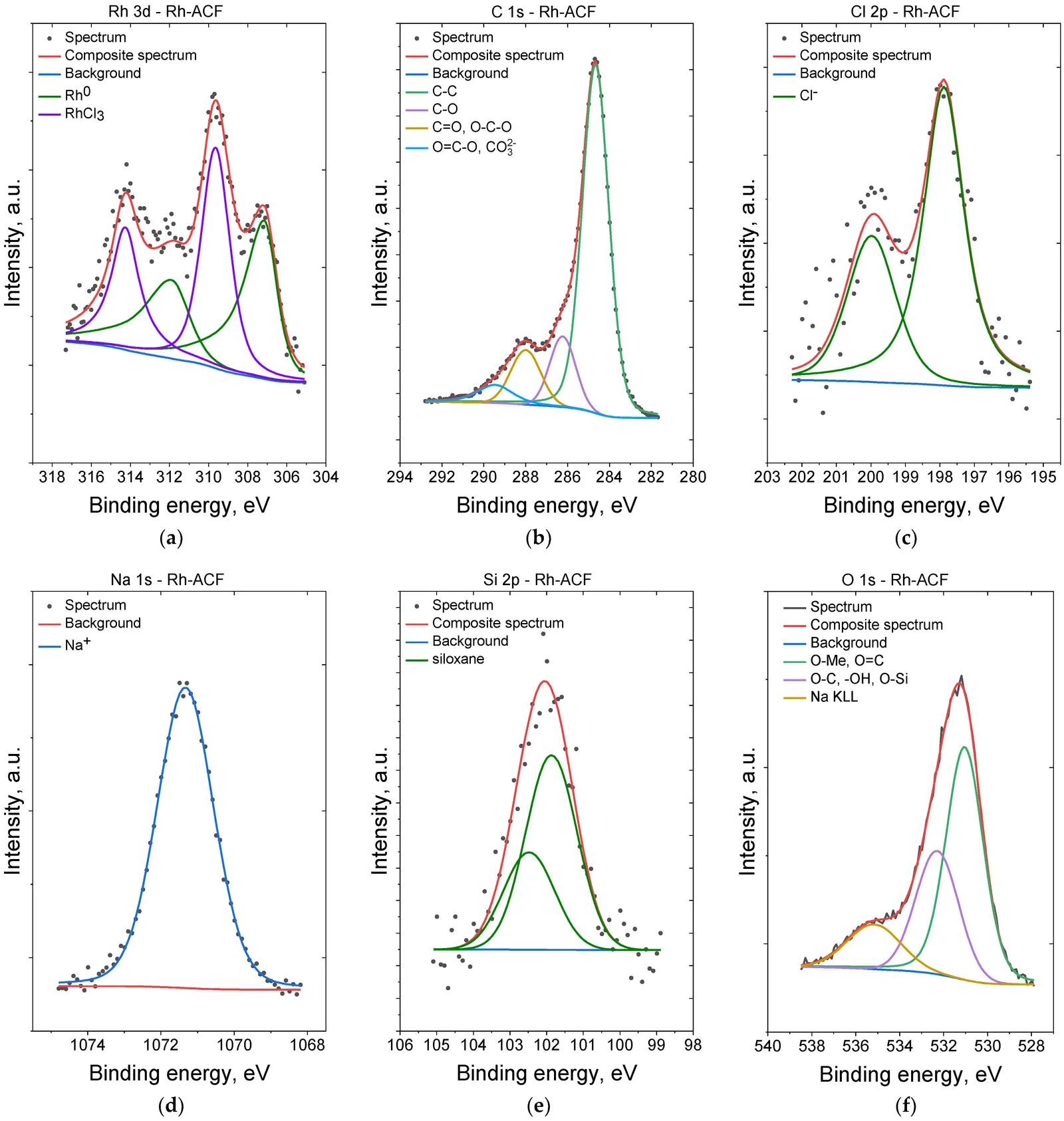
XPS spectra of the Rh@ACF catalyst: Rh 3d (**a**), C 1s (**b**), Cl 2p (**c**), Na 1s (**d**), Si 2p (**e**), and O 1s (**f**).

Surface concentrations of chemical bonds obtained from fitting XPS data (Figure 6) for all samples are listed in Table 1.

For the sample Rh-ACF the Rh 3d spectrum was fitted with two doublet structures (d5/2–d3/2 doublet separation equals 4.75 eV) with first 3d5/2 line centered at 307.2 eV, which indicates the presence of metallic rhodium, and a second 3d5/2 line centered at 309.8 eV, which originates from the presence of RhCl_3_ [35,36,37,38]. The C 1s spectra for both samples were fitted with four components. The first line centered at 285.0 arises from aliphatic carbon C-C-type bonds, a second line lies at 286.5 eV and indicates the presence of C-O and/or C-OH bonds, a third line centered at 288.2 eV indicates the presence of C=O and/or O-C-O bonds [39,40], and a fourth line at 289.7 eV indicates the presence of CO_3_^2−^-type compounds and/or O-C=O bonds [39]. The O 1s spectra for both samples were fitted with three components with slight differences in binding energies: a first line centered at 531.0 eV which indicates mainly oxygen in metal oxides and O=C organic type bonds, a second line at 532.3 eV indicating defective oxygen in metal oxides and/or O-Si bonds and/or –OH groups or organic species containing O-C-type bonds. A third line, found at ~535–536 eV, comes from the Auger Na KLL process [35,41,42]. The Si 2p spectrum for Rh-ACF sample shows one doublet structure (doublet separation p3/2–p1/2 equals 0.61 eV) with main 2p3/2 line centered at 102.1 eV which indicate silicone or siloxane type compounds [35,43]. The Cl 2p spectra were fitted with a doublet structure (doublet separation p3/2–p1/2 equals 1.6 eV) with a main 2p3/2 line centered at 197.9 eV which indicates the presence of Cl¯ ions in chlorides (both NaCl and RhCl_3_) [35]. The Na 1s spectra for both samples were fitted with single line centered at 1071.3 eV which indicates the presence of Na^+^ ions [44].

### 3.8. Catalytic Test Performance for 4-NP Reduction to 4-AP in the Batch Reactor

**Figure 7:**
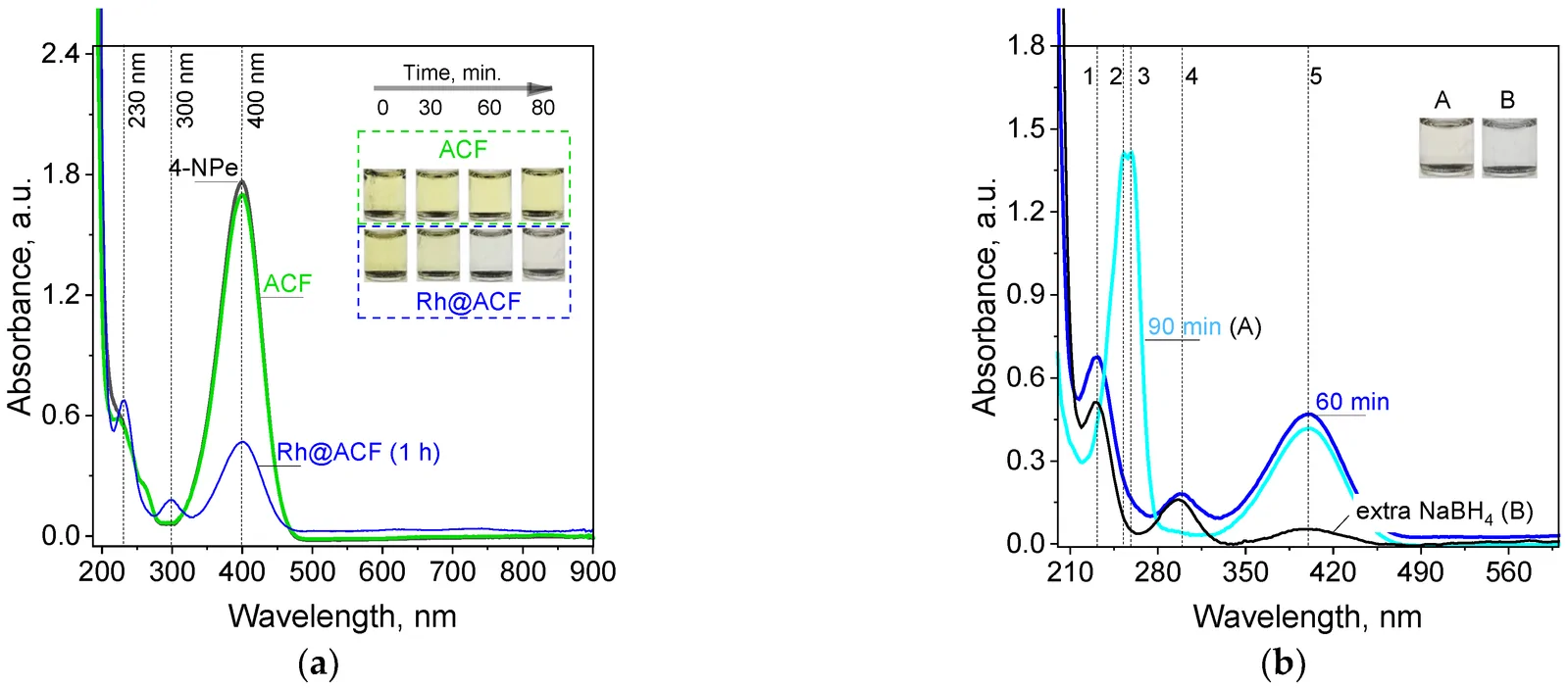
The color evolution as well as UV-Vis spectra registered for solutions containing only 4-NPe and with ACF (catalyst carrier) or Rh@AFC (catalyst) addition, respectively (**a**), spectra evolution and colors change during longer time (A) and extra NaBH_4_ powder addition (B) to the reacting mixture (**b**). Conditions: m_ACF_ = m_Rh@ACF_ = 0.001 g, V = 3 mL, T = 20 °C, time of the process 90 min. Peaks assignments: 1–230, 2–253, 3–258, 4–300 (295, B), and 5–400 nm.

Catalytic tests were performed for model reaction, i.e., the reduction of 4-nitrophenol (4-NP) to 4-aminophenol (4-AP). This process was carried out in the batch reactor and it has two steps. The first one relates to conversion of 4-NP to its conjugate form, i.e., 4-nitrophenolate (4-NPe), which is formed immediately after NaBH_4_ addition. This process is very fast and easy to observe by the naked eye due to a clear color change in the reacting mixture from slightly to clearly yellow. The obtained product also has a characteristic UV-Vis spectrum with a strong maximum at 400 nm (Figure 7a).

**Figure 8:**
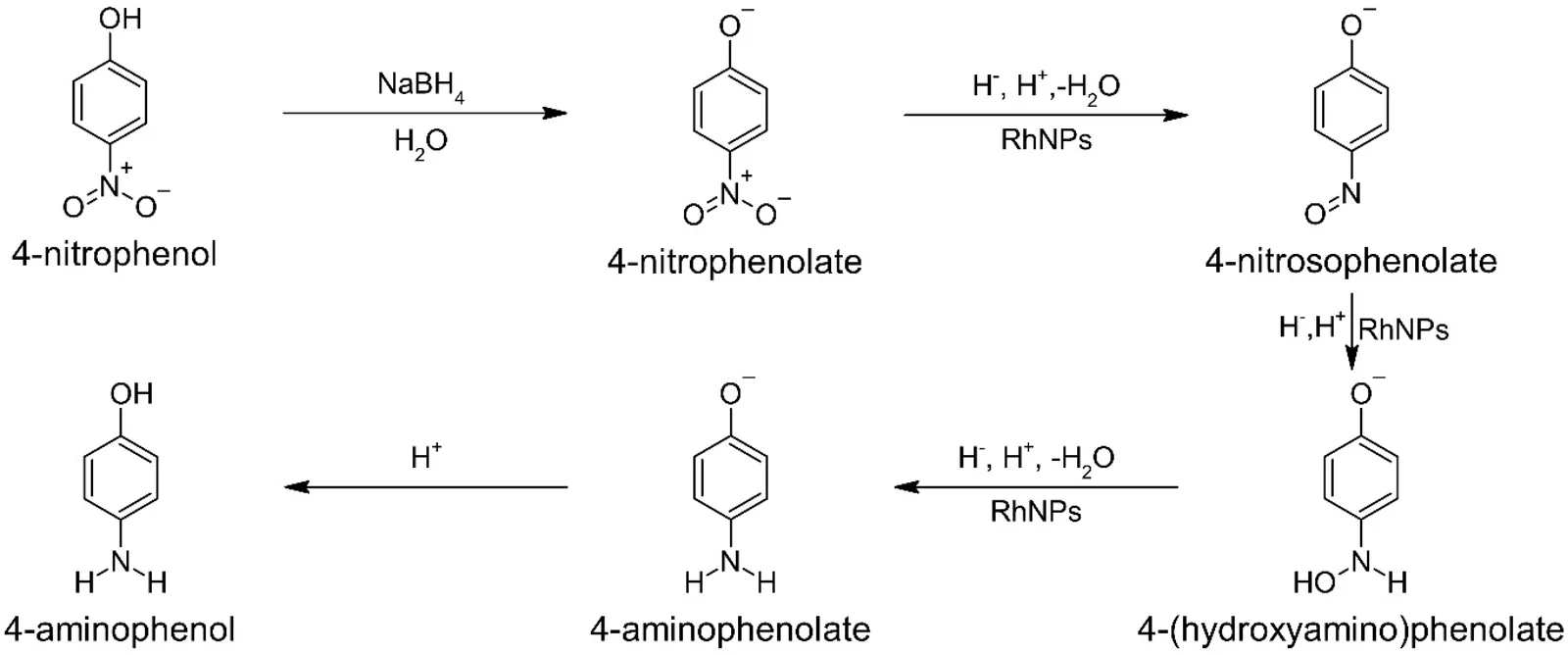
A schematic representation of the mechanism illustrating the conversion of 4-nitrophenol to 4-aminophenol, including the most probable intermediates.

The location of the maximum is in good accordance with literature data [45] and confirms the presence of 4-NPe in the solution. Then, this solution was used for catalytic tests. For this purpose 3 mL of aqueous mixture containing 4-NPe was added to 1.0 mg of ACF or Rh@ACF. Both carbon structures were well dispersed in the solution by shaking the vials. The ACF addition to 4NPe did not cause any visible changes in the color of the sample as well in the characteristic spectrum (Figure 7a). The percentage change in the absorbance value registered at 400 nm equals about 4%, 1h later. For comparison, during process, in which a catalyst was present, the yellow color coming from 4-NPe changed to slightly yellow (Figure 7a,b, A) and finally to colorless after the addition of an extra portion of NaBH_4_ powder (Figure 7b, B). The colorless solution suggests 4-aminophenol formation and a characteristic UV-Vis spectrum with maxima at 230 and 300 nm [45,46]. Thus, the catalytic effect confirmed the catalytic properties of Rh nanoparticles deposited on ACFs. Due to the course of the catalytic reaction, we observed that the process was somehow stopped (Figure 7b). Moreover, the spectrum previously characteristic for 4-AP changed (disappearance of the maximum at 300 nm, A), a new maxima were formed which apart from signals from an unreacted 4-NPe at 400 were detected at 258 and 295 nm. These double peaks have not previously been reported. These peaks can be assigned to the 4-(hydroxyamino)phenolate (Figure 8), which is a derivative of phenol. Phenolic compounds generally exhibit UV-Vis absorption bands in the 270–300 nm region due to π-π* transitions within the aromatic ring.

The obtained data confirmed the catalytic properties of RhNPs deposited on ACFs. The full conversion of 4-NPe took place but after the addition of an extra portion of sodium borohydride, which forwarded the process. The conversion reached 97% 5 min later, indicating high catalytic performance. Considering the small amount of Rh@ACF used in this experiment (i.e., 0.001 g), we performed precise calculations to determine the quantity of catalyst introduced into the catalytic reaction. Each individual process involved only 4.24 µg of RhNPs per sample (volume = 3 mL). This demonstrates exceptional catalytic efficiency, comparable to that of similar materials [38]. Moreover, the cost of the catalyst used per test is less than 0.01 PLN (based on a price of 2181.13 PLN per gram, source: Mennica Państwowa, Warszawa, Poland), even under the assumption of single-use application. These findings indicate that the developed method for catalyst production is highly promising and may be suitable for metal recovery from waste solutions.

## 4. Conclusions

The research conducted and the results obtained demonstrate that the microreactor system is a highly effective tool for synthesizing 3–4 nm rhodium nanoparticles and depositing them onto activated carbon fibers in a single step. This approach shows great promise for future scale-up and for potential applications in recovering metal ions directly from waste solutions into ready-to-use catalysts. The synthesized catalyst, Rh@ACF, exhibited excellent performance in the reduction of 4-nitrophenolate to 4-aminophenol, highlighting the critical role played by sodium borohydride in the reaction mixture. However, the results also revealed that prolonged catalysis (1 h) was hindered by an insufficient amount of substrate, specifically NaBH_4_. On the other hand, excessive amounts of NaBH_4_ should be avoided due to two interrelated concerns. First, rhodium actively promotes the decomposition of NaBH_4_, which leads to rapid hydrogen release from the solution and inefficient use of reagents, as not all are consumed in the catalytic process. Second, from an environmental perspective, using large quantities of this toxic and mutagenic reagent is neither safe nor sustainable. Therefore, a two-step addition of sodium borohydride appears to be more rational than a single-step approach, as it enables optimal utilization of the reagent.

## Figures and Tables

**Table 1:** Surface composition (atomic %) determined by fitting XPS data.

	C				O		Na	Si	Cl	Rh	
Binding energy [eV]	285.0	286.5	288.2	289.7	531.0	532.3	1071.3	102.1	197.9	307.2	309.6
Groups/Ox. State	C-C C-H	C-O C-OH	C=O O-C-O	O-C=O CO_3_^2−^	O-Me O=C	O-C-OH O-Si	Na^+^	siloxane silicone	Cl^−^	Rh^0^	RhCl_3_
Rh-ACF	43.5	7.9	7.0	3.4	17.4	9.7	8.1	1.6	0.4	0.4	0.7

## Data Availability

The original contributions presented in this study are included in the article/supplementary material. Further inquiries can be directed to the corresponding author.

## Supplementary Materials

The following supporting information can be downloaded at: https://www.mdpi.com/article/10.3390/nano15171375/s1, Figure S1: Sample of kinetic curve registered for process of complex formation between Rh (III) and ethanol in basic medium at 30 °C; Figure S2: Results of EDS chemical analysis of Rh@ACF (a’–e’) performed in the areas marked on the SEM image (a–e); Table S1: EDS chemical analysis of obtained catalyst in different areas (weight, %).
