# Supplementary material for: One-Step Synthesis of Ultra-Small RhNPs in the Microreactor System and Their Deposition on ACF for Catalytic Conversion of 4–Nitrophenol to 4–Aminophenol
Source: Nanomaterials (Basel). 2025 Sep 5;15(17):1375. doi: 10.3390/nano15171375 (PMC12429861; doi:10.3390/nano15171375)
Supplement: Supplementary file 1 [file nanomaterials-15-01375-s001.zip › nanomaterials-3785914-supplementary.pdf]

Supporting Materials

One-Step Synthesis of Ultra-Small RhNPs in the Microreactor System and Their Deposition on ACF for Catalytic Conversion of 4–Nitrophenol to 4–Aminophenol

Adrianna Pach, Konrad Wojtaszek, Ahmed Ibrahim Elhadad, Tomasz Michałek, Anna Kula

AGH University of Krakow, Faculty of Non-Ferrous Metals, Al. A, Mickiewicza 30, 30-059 Kraków, Poland; apach@agh.edu.pl (A.P.); kwojtasz@agh.edu.pl (K.W.); elhadad@agh.edu.pl (A.I.E.); tomaszm@agh.edu.pl (T.M.); kula@agh.edu.pl (A.K.)

* Correspondence: mlb@agh.edu.pl

S1. Process of complex formation between Rh(III) and ethanol in basic medium

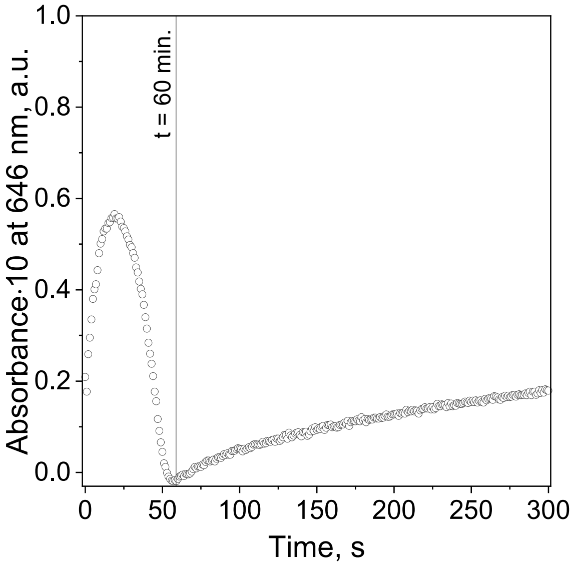

Figure S1. Sample of kinetic curve registered for process of complex formation between Rh(III) and ethanol in basic medium at 30°C.

S2. EDS analysis of Rh@ACF

C Rh(1)

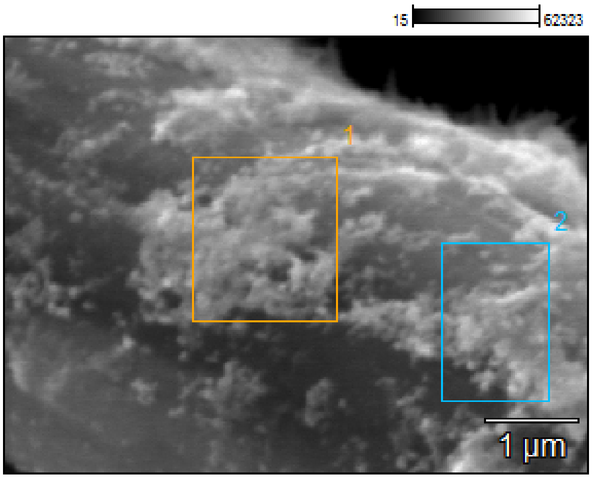

(a)

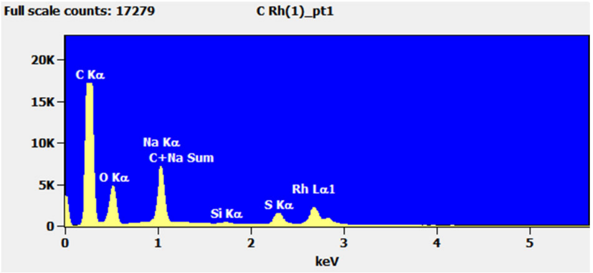

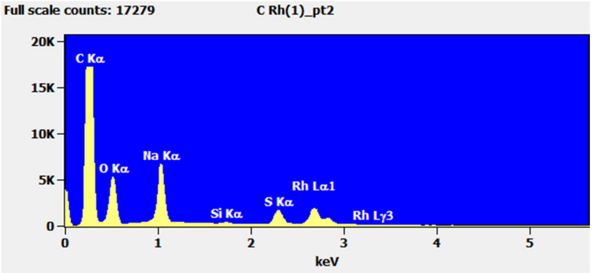

(a’)

C Rh_1

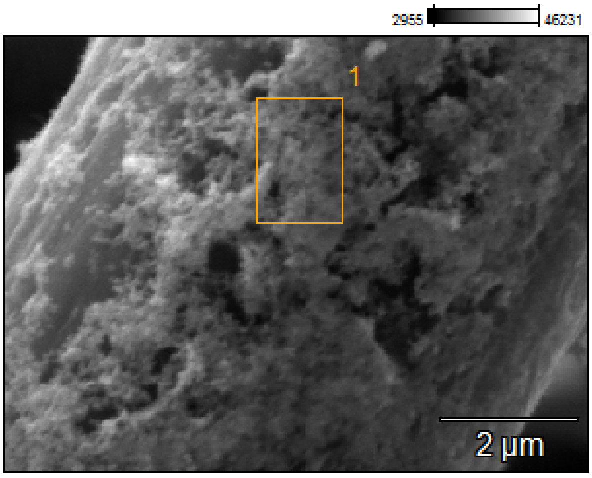

(b)

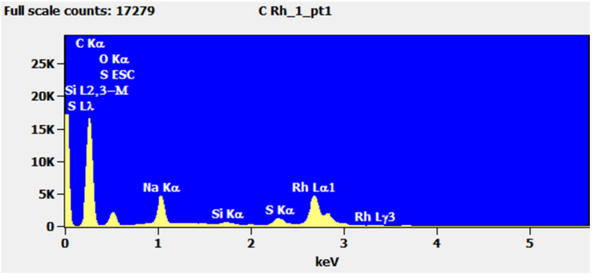

(b’)

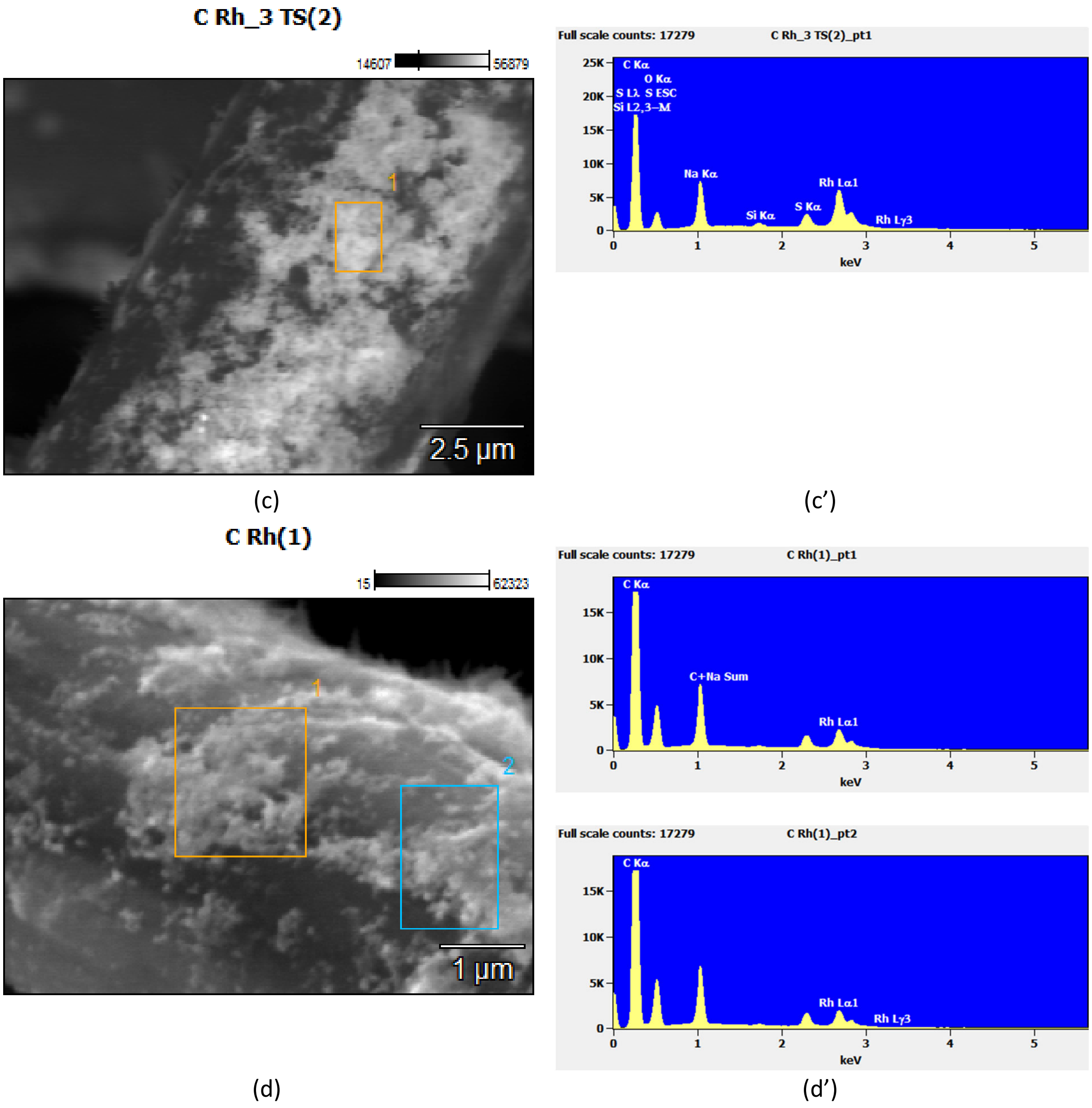

Figure S2. Results of EDS chemical analysis of Rh@ACF (a’– e’) performed in the areas marked on SEM image (a – e).

Table S1. EDS chemical analysis of obtained catalyst at different area (weight, %).

Sample	C-K	O-K	Na-K	Si-K	S-K	Rh-L
C Rh(1)_pt1	60.16	24.58	7.40	0.13	1.41	6.32
C Rh(1)_pt2	61.25	25.43	6.62	0.11	1.42	5.17
C Rh_1_pt1	52.13	17.88	7.59	0.43	1.58	20.40
C Rh_3 TS(2)_pt1	58.90	19.64	8.20	0.33	1.43	11.52
C Rh(1)_pt1	82.52					17.48
C Rh(1)_pt2	61.25	25.43	6.62	0.11	1.42	5.17
